# H3.3 demarcates GC-rich coding and subtelomeric regions and serves as potential memory mark for virulence gene expression in *Plasmodium falciparum*

**DOI:** 10.1038/srep31965

**Published:** 2016-08-24

**Authors:** Sabine Anne-Kristin Fraschka, Rob Wilhelmus Maria Henderson, Richárd Bártfai

**Affiliations:** 1 Department of Molecular Biology, Radboud University, Nijmegen, the Netherlands

## Abstract

Histones, by packaging and organizing the DNA into chromatin, serve as essential building blocks for eukaryotic life. The basic structure of the chromatin is established by four canonical histones (H2A, H2B, H3 and H4), while histone variants are more commonly utilized to alter the properties of specific chromatin domains. H3.3, a variant of histone H3, was found to have diverse localization patterns and functions across species but has been rather poorly studied in protists. Here we present the first genome-wide analysis of H3.3 in the malaria-causing, apicomplexan parasite, *P. falciparum*, which revealed a complex occupancy profile consisting of conserved and parasite-specific features. In contrast to other histone variants, *Pf*H3.3 primarily demarcates euchromatic coding and subtelomeric repetitive sequences. Stable occupancy of *Pf*H3.3 in these regions is largely uncoupled from the transcriptional activity and appears to be primarily dependent on the GC-content of the underlying DNA. Importantly, *Pf*H3.3 specifically marks the promoter region of an active and poised, but not inactive antigenic variation *(var)* gene, thereby potentially contributing to immune evasion. Collectively, our data suggest that *Pf*H3.3, together with other histone variants, indexes the *P. falciparum* genome to functionally distinct domains and contribute to a key survival strategy of this deadly pathogen.

In all eukaryotes, chromatin enables high-degree compaction of the genetic material while allowing access to the DNA for essential cellular processes such as transcription, replication, DNA damage response and repair. Canonical histones, namely H2A, H2B, H3 and H4, are mainly synthesized and assembled into nucleosomes in a replication-coupled manner to form the basic layout of the newly synthetized chromatin^1^,^2^,^3^. In contrast, histone variants (non-allelic histone isoforms^1^,^3^), and most posttranslational histone modifications (e.g. acetylation, methylation^4^) are locally incorporated into chromatin in a replication-independent fashion to alter its properties and function.

Given their essential function, histones are among the most conserved proteins and their evolutionary origins can be traced back to archaea, where one or two histone like proteins are present^5^. In Eukarya, the histone family has expanded into canonical histones, the linker histone H1 and a great variety of histone variants for H2A, H2B and H3^6^. Strikingly, the amino acid sequences of the four eukaryotic canonical histones and of many histone variants are extremely similar among distantly related species, although histone variants have emerged by convergent evolution^7^,^8^,^9^. The requirement of convergent evolution of histone variants indicates a universal theme in chromatin regulation; and indeed some histone variants display universal functions. Histone H3 variant CenH3, for instance, demarcates the centromere and functions in chromosome segregation^10^,^11^,^12^,^13^. The function of H3.3, another member of the H3 histone family is however more diverse among the species.

Phylogenetically earlier organisms, such as *Saccharomyces cerevisiae* and the algal protist, *Cyanidioschyzon merolae*, possess only one form of non-centromeric H3 that is needed for both replication-coupled and replication-independent assembly^11^,^14^. This single form corresponds to variant H3.3 of higher eukaryotes, which suggests that canonical forms of H3 evolved recurrently in eukaryotic evolution, most likely by divergence from H3.3-like forms^15^,^16^. In most eukaryotes H3.3 differs from H3 only by few amino acid substitutions; for example by four in most mammals and plants and by 16 in the ciliate *Tetrahymena thermophila* (Superphylum Alveolata)^11^,^17^. Yet, these few amino acid substitutions are sufficient to determine H3.3 and H3 chaperone selectivity and their respective nucleosome deposition pathways^18^,^19^,^20^,^21^.

Genome-wide studies performed mainly in animals, but also in *Arabidopsis thaliana* revealed that H3.3 is mainly deposited into the coding sequence of transcribed genes, promoters of active and inactive genes, transcription start and end sites as well as further gene regulatory elements in euchromatic regions^8^,^16^,^22^,^23^. H3.3 is incorporated into transcription start sites and coding regions of genes in a transcription-coupled manner^24^,^25^. However, enrichment of H3.3 has also been observed in regions of the genome that are presumed to be transcriptionally silent^26^,^27^,^28^,^29^,^30^. For example, in embryonic stem cells H3.3 contributes to the maintenance of the condensed chromatin state of telomeres^26^,^28^,^31^,^32^, while it appears to be absent from the telomeres of *A. thaliana*^33^,^34^. Surprisingly, deletions of H3.3 genes are not always lethal, probably due to compensation by other H3 genes. For instance, increased H3 expression compensates for H3.3 lacking in *D. melanogaster*^35^ and overexpression of H3.3 makes canonical H3 dispensable in *T. thermophila*^17^. In contrast, H3.3 disruption in *D. melanogaster* and in *T. thermophila* results in meiotic defects^17^,^36^,^37^. Furthermore, in mammals, H3.3 is not only required for reproduction but also for early development^32^,^38^ and mutation in H3.3 can lead to various pediatric cancer types^39^,^40^. Hence, the function of H3 variants has been extensively studied in animals, fungi and plants - where it appears to have several conserved, but also specialized function.

In this article, we focus on the localization and role of H3.3 in the apicomplexan parasite, *Plasmodium falciparum*. Besides the canonical histones four histone variants are encoded in the *Plasmodium* genome, i.e. *Pf*H2A.Z, *Pf*H2B.Z, *Pf*CenH3 and *Pf*H3.3^41^,^42^. Interestingly, *P. falciparum* has an extremely AT-rich genome (on average ~80% adenine and thymine bases) where different genomic regions have distinctly different base composition. Earlier we observed an intriguing correlation between the base composition of these genomic regions and the incorporation of various histone variants^41^. Centromeres are formed via incorporation of *Pf*CenH3 into a ~2kb long extremely AT-rich (~97% AT) sequence present once on every chromosome^13^. On the other end of the spectrum are H3K9me3 and HP1-marked heterochromatic regions with distinctly higher GC content (~73% AT). These regions are present at the chromosome ends and some chromosome internal islands, and so far have found to contain mainly canonical nucleosomes^43^,^44^,^45^,^46^. Within the euchromatic domain intergenic regions having distinctly higher AT-content (~87% AT) compared to coding sequences (~78% AT), are demarcated by double-variant nucleosomes containing *Pf*H2A.Z and the *Plasmodium*-specific variant *Pf*H2B.Z^46^,^47^,^48^,^49^. Collectively these observations support a model in which histone variants help indexing the *Plasmodium* epigenome into functionally distinct domains and the rough blueprint of the epigenome is sketched by the AT-content of the underlying sequence. Here, in further support of this model, we show that *Pf*H3.3 demarcates euchromatic coding sequences and subtelomeric repeat regions in a GC-content coupled-manner. Furthermore, we observe that *Pf*H3.3 specifically associates with the promoter region of the single expressed or poised - but not inactive - antigenic variation gene (*var* gene). This suggests that *Pf*H3.3 might contribute to the epigenetic memory of *var* gene expression and hence could underlie a major defense mechanism of this deadly human pathogen.

## Results

### *Pf*H3.3 primarily localizes to GC-rich euchromatic coding sequences and subtelomeric repetitive regions

*Plasmodium falciparum*’s histone variant *Pf*H3.3 (*PF*3D7_0617900) and its single copy canonical counterpart *Pf*H3 (*PF*3D7_0610400) both encode for a 136 amino-acid protein. They only differ in eight amino acids including an Apicomplexan specific feature for *Pf*H3.3, namely the substitution of the conserved amino acids RY with KF at position 54 and 55^42^,^50^. With an identity of 94% they are hence difficult to investigate individually employing antibodies.

We circumvented this problem by generating a *P. falciparum* 3D7 parasite line that expresses an episomal Ty1-tagged copy of *Pf*H3.3 under the control of the chloroquine resistance transporter promoter (similar to pARL-1a-Ty1-H2A in ref. 47). Additionally, to avoid any artefacts due to episomal (over)expression of the tagged protein, we engineered a construct (pHH1-*Pf*H3.3-Ty1) that, after single crossover integration, resulted in a *P. falciparum* NF54-DCJ parasite line^51^ expressing *Pf*H3.3-Ty1 under control of the endogenous promoter (Fig. S1). Notably, these tagged lines show a very similar multiplication rate (data not shown) and expression pattern (Fig. S2) to wild type parasites suggesting that the tag does not interfere with normal function of *Pf*H3.3.

To determine the genome-wide localization of *Pf*H3.3, we performed chromatin immunoprecipitation of mono-nucleosomes using an anti-Ty1 antibody followed by next generation sequencing of the immunoprecipitated DNA (ChIP-seq). First we performed a test ChIP-seq on native chromatin isolated from the episomal Ty1-*Pf*H3.3 line for two intraerythrocytic stages (30 and 40 hours post-invasion) in duplicates (Fig. S3). The respective ChIP input samples were sequenced as control. For detailed analysis we performed ChIP-seq on formaldehyde cross-linked chromatin from the endogenously *Pf*H3.3-Ty1 line for four intraerythrocytic stages (10, 20, 30 and 40 hpi). Additionally, we performed ChIP-seq using an anti-H3 antibody that recognizes the C-terminal end of both *Pf*H3.3 and *Pf*H3 (referred to as H3core control) and RNA-seq on the same four parasite populations. The resulting *Pf*H3.3 ChIP-seq data were normalized either to input or H3core ChIP-seq data to correct for any biases due to differential cross-linking, PCR amplification efficiency and reduced mapability of the highly AT-rich sequences, typical to the *P. falciparum* genome^47^. The normalized *Pf*H3.3 ChIP-seq data from episomal and endogenously *Pf*H3.3-tagged parasites displayed a highly similar localization pattern (Fig. S3A) and good genome-wide correlation (Fig. S3B; r = 0.71–0.86), despite numerous technical differences between the two experiments (N- versus C-terminal tagging, cross-linked versus native ChIP, different parasite lines, different library preparation methods) (Fig. S3). Hence, our ChIP-seq analysis provides a robust measure of *Pf*H3.3 localization, which we analyzed in detail using the data from the endogenously tagged line.

**Figure 1:**
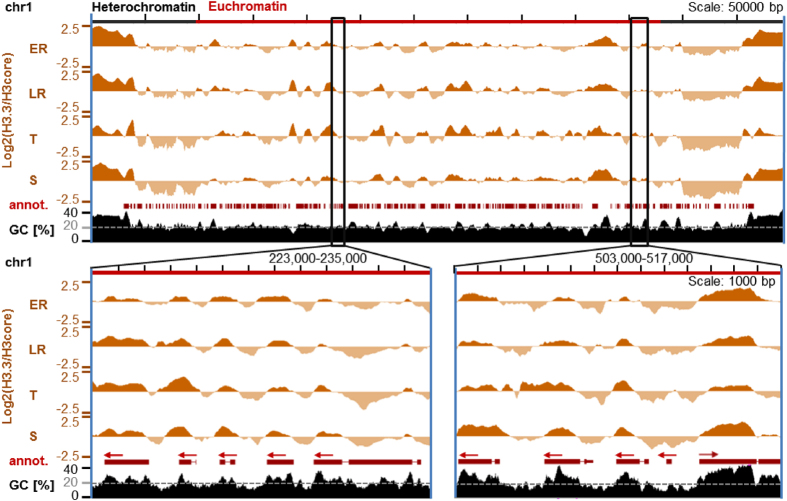
*Pf*H3.3-Ty1 ChIP-seq profiles from different stages of intraerythrocytic development. ChIP-seq ratio tracks displaying relative enrichment of the endogenous *Pf*H3.3-Ty1 compared to H3core (antibody capturing histone *Pf*H3 and *Pf*H3.3) over entire chromosome 1 (upper panel) or two smaller sections of it (lower panels). Red arrows indicate the directionality of the respective gene (“annot.”). Stages: ER: early rings (10 hpi), LR: late rings (20 hpi), T: trophozoites (30 hpi), S: schizonts (40 hpi). Heterochromatic regions are defined based on HP1 occupancy in an earlier study^43^.

*Pf*H3.3 is mainly enriched in euchromatic coding sequences and is generally absent in the heterochromatic domain with the exception of its enrichment over GC-rich subtelomeric repetitive sequences (Fig. 1). In these regions the occupancy of *Pf*H3.3 is largely invariable through the intraerythrocytic stages. Additionally, *Pf*H3.3 occupancy shows dynamic changes in euchromatic intergenic regions (Fig. 1). Centromeres are depleted of *Pf*H3.3 and *Pf*H3 (Fig. S4), as they are occupied by the third H3 variant, *Pf*CenH3^13^.

### *Pf*H3.3 is demarcating the *P. falciparum* genome and exogenous DNA sequences in a GC-content coupled manner

Visual inspection of the genome-wide *Pf*H3.3 profiles led to the observation that *Pf*H3.3 is mainly incorporated into GC-rich sequences (Fig. 1), including euchromatic coding sequences with an average GC-content of ~22% and the subtelomeric repetitive sequences with an GC-content ~32%. While at these GC-rich regions *Pf*H3.3 occupancy is invariable across the stages, AT-rich intergenic regions (average 14% GC) are only temporarily marked or completely depleted of *Pf*H3.3 (Fig. 1).

**Figure 2:**
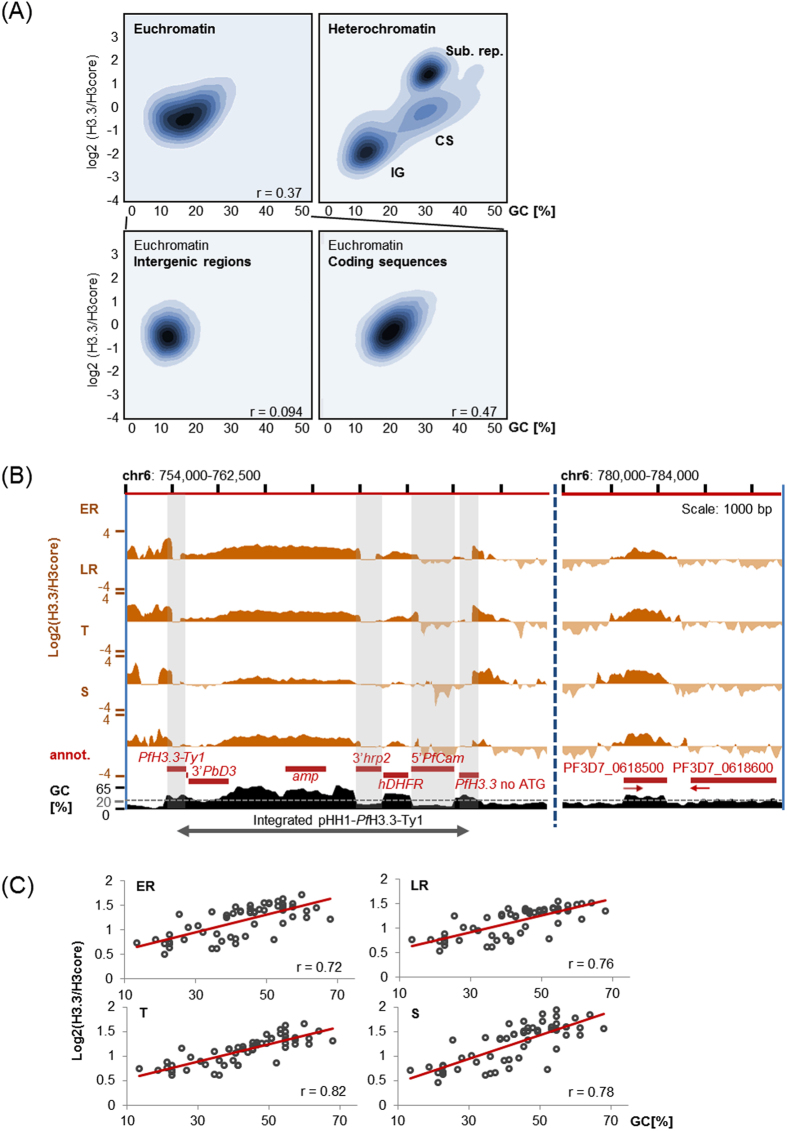
*Pf*H3.3 localizes to GC-rich regions within the *P. falciparum* genome and exogenous DNA. (**A**) Density plots depicting *Pf*H3.3 levels in relation to GC-content. *Pf*H3.3 levels and GC-content were calculated genome-wide per 150 bp windows. Data depicted are generated from schizont stages. IG: intergenic region, CS: coding sequence, Sub. Rep.: subtelomeric repetitive region. Pearson correlation values are displayed at the lower right corner of the graphs. (**B**) Screen shot from *Pf*H3.3 ChIP-seq ratio tracks encompassing the integrated pHH1-*Pf*H3.3-Ty1 plasmid sequence (left) and the sequence of two nearby genes (right). Grey blocks indicate non-unique *P. falciparum* sequences within the pHH1-*Pf*H3.3-Ty1 plasmid, which were excluded from the analysis in (**C**). (**C**) Scatter plot of GC percentage and *Pf*H3.3 occupancy over unique 75 bp windows of the integrated pHH1-*Pf*H3.3-Ty1 plasmid. Stages: ER: early rings (10 hpi); LR: late rings (20 hpi), T: trophozoites (30 hpi), S: schizonts (40 hpi). Pearson correlation values are displayed at the lower right corner of the graphs.

To further investigate the correlation between GC-content and *Pf*H3.3 occupancy on a genome-wide level, we *in silico* subdivided the genome into 150 bp windows and calculated the GC percentage and *Pf*H3.3 coverage for each window. In line with our visual observation, GC-poor intergenic regions show the lowest *Pf*H3.3 coverage; whereas in euchromatic coding sequences *Pf*H3.3 levels exhibited clear correlation with the GC-content (Fig. 2A and Fig. S5A for data from all four stages). Importantly, similar correlation was also observed in the ChIP-seq data obtained from the episomally tagged line (Fig. S5B). Subtelomeric GC-rich repetitive sequences display the highest *Pf*H3.3 levels. Thus, our genome-wide computational analysis is consistent with the initial observation.

Additionally, we tested whether *Pf*H3.3 is also incorporated into an exogenous DNA in a GC-dependent manner. Therefore, the non-*P. falciparum*-specific sequences within the integrated pHH1-Ty1-*Pf*H3.3 plasmid were examined for their GC-content and *Pf*H3.3 occupancy. Also here a clear correlation of incorporated *Pf*H3.3 and GC-content can be observed (Fig. 2B,C). As these exogenous sequences are unlikely to contain sequence-element for recruitment or detainment of *Pf*H3.3 or its chaperone, these findings support a base-content-driven genome marking by *Pf*H3.3.

### *Pf*H3.3 dynamically occupies intergenic regions predominantly during trophozoite development

**Figure 3:**
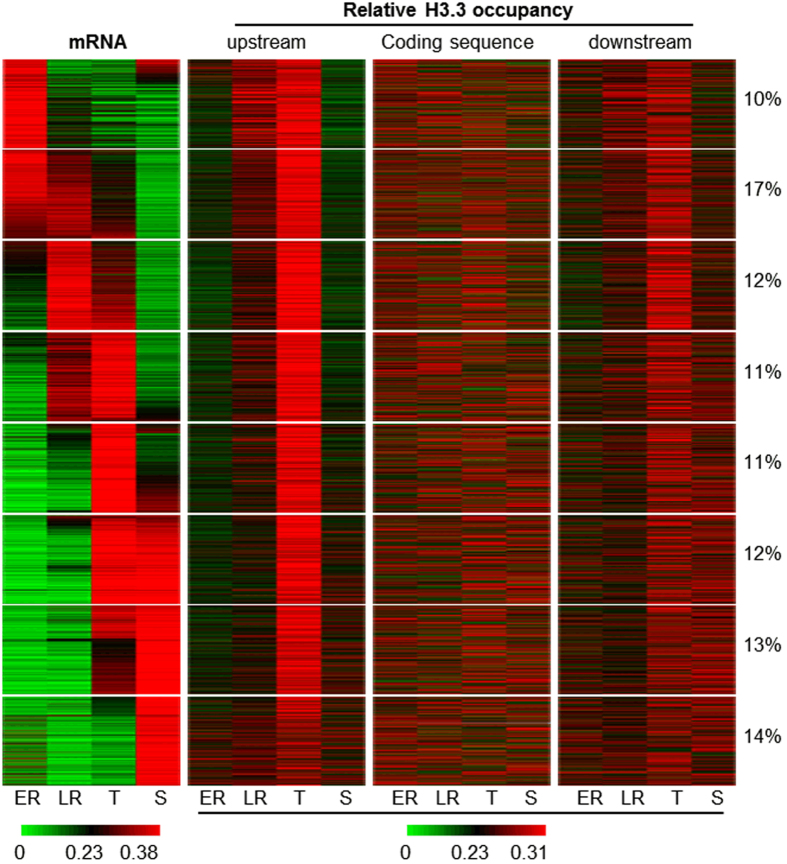
*Pf*H3.3 dynamically occupies intergenic regions predominantly during trophozoite stage. Heatmap represents relative mRNA abundance of 3800 genes (heterochromatic and genes with low mRNA abundance have been excluded) and relative *Pf*H3.3 occupancy in upstream, coding and downstream region throughout intraerythrocytic development. Color-scale depicts the relative mRNA abundance in relation to the sum of the RPKM values in all four stages or the relative PfH3.3 occupancy in proportion to the sum of occupancy values in all four stages (green – low, red – high). 8 groups of co-expressed genes were identified using k-means clustering. The percentage of genes belonging to each cluster is displayed next to the heatmap. Stages: ER: early rings (10 hpi), LR: late rings (20 hpi), T: trophozoites (30 hpi), S: schizonts (40 hpi).

The dynamic occupancy of H3.3 in some intergenic regions prompted us to test whether correlation exists between temporal gene activity and the dynamics of *Pf*H3.3 occupancy. Genes were clustered according to their relative mRNA abundance (matching RNA-seq data) during the intraerythrocytic cycle and matched with the respective *Pf*H3.3 occupancy values (in proportion of the sum of the occupancy values) for upstream regions, coding sequences and downstream regions (Fig. 3). Whereas *Pf*H3.3 coverage in coding sequences showed no obvious stage-dependent or transcription-coupled dynamics, mainly upstream regions but to some extent also downstream regions showed dynamic *Pf*H3.3 enrichment during the intraerythrocytic cycle. Interestingly, most intergenic regions have the highest relative *Pf*H3.3 occupancy during the trophozoite stage largely independent of their transcriptional dynamics.

To investigate *Pf*H3.3 enrichment in relation to gene expression level, we plotted the mRNA abundance (RPKM values) for each gene against *Pf*H3.3 occupancy in coding, upstream or downstream regions for each stage separately (Fig. S6). This analysis also did not reveal clear correlations between transcription and *Pf*H3.3 occupancy. Only in 5′ intergenic regions and in particular at the trophozoite stage were moderate correlations observed.

In summary, *Pf*H3.3 is temporarily enriched in AT-rich, 5′ intergenic regions mainly at the trophozoite stage. While this enrichment is somewhat more apparent for very highly expressed genes, and least apparent for (nearly) silent genes (Fig. S6), it does not exhibit clear correlation to gene expression dynamics (Fig. 3).

### *Pf*H3.3 marks the promoter of the active and poised var gene

Persistence of *P. falciparum* within the human bloodstream primarily depends on the *var* multigene family, encoding for a highly variable cytoadherence protein called *P. falciparum* erythrocyte membrane protein 1 (*Pf*EMP1)^52^,^53^,^54^,^55^. Only one of the ~60 *var* gene family members is expressed in any given parasite while other family members remain transcriptionally silenced. Importantly, *var* genes are expressed in a clonally variant fashion^56^ as the same *var* gene is reactivated over several generation by a yet elusive mechanisms of epigenetic memory^57^,^58^.

**Figure 4:**
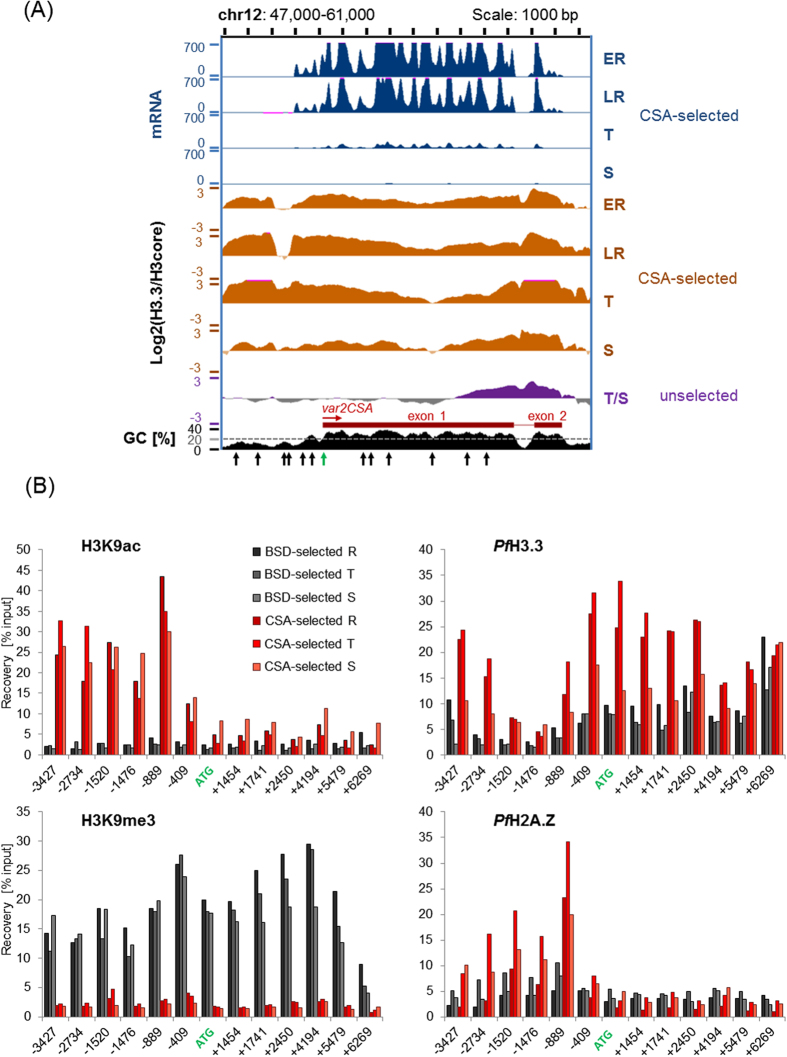
*Pf*H3.3 marks the promoter of the active and poised, but not the inactive *var* gene. (**A**) RNA-seq and ChIP-seq ratio tracks displaying *Pf*H3.3 enrichment over H3core over the *var*2CSA locus. Data was obtained from *var2CSA*-selected parasites at four intraerythrocytic stages (ER, LR, T, S) as well as from unselected mixed stage parasites (mainly containing trophozoites and schizonts, T/S). Arrows indicate the approximate position of primers used in (**B**). (**B**) Bar graphs displaying ChIP-qPCR data for H3K9ac (euchromatic mark) and H3K9me3 (heterochromatic mark) marking as well as *Pf*H3.3 and *Pf*H2A.Z occupancy over the *var2*CSA locus in CSA-selected (red) or - BSD-selected (grey) parasites. After affinity-based selection for *var*2CSA-expressing parasites half of the parasite population was forced to express PF3D7_0223500 by blasticidin treatment (NF54-DCJ strain: first exon of PF3D7_0223500 is replaced by a blasticidin resistance cassette). Numbers indicate the position of the forward primer relative to the ATG. Stages (R: rings (15 hpi); T: trophozoites (25 hpi); S: schizonts (40 hpi)) are depicted by different shades.

In order to investigate the role of *Pf*H3.3 in *var* gene regulation, we specifically looked at the *var2CSA* locus of CSA-selected (10, 20, 30, 40 hpi) (Fig. S7A) and non-selected (mix of trophozoites and schizonts) parasites. Interestingly, *Pf*H3.3 is enriched in the promoter as well as in the coding region of the active as well as poised *var2CSA* gene (Fig. 4A) (Note that the *var* gene is only transcribed during rings stages while at trophozoites and schizonts stages it is poised, yet marked for reactivation in the next generation). The *var2CSA* gene in non-CSA selected parasites, however, is clearly less enriched in *Pf*H3.3 and only shows *Pf*H3.3 coverage at the *var2CSA* intron and the second exon (regions which are known to be transcribed from the *var* intronic promoter^55^,^59^,^60^,^61^,^62^,^63^) (Fig. 4A). To ensure that this observation was not an artefact due to the comparison of two independently generated data sets (as the CSA-selected and non-selected parasites originate from independent experiments) we performed a ChIP-qPCR experiment that enabled us to directly compare the *var2CSA* locus of CSA-selected and non-selected parasites. First, a culture of endogenously *Pf*H3.3-Ty1 expressing NF54-DCJ *P. falciparum* parasites was selected for *var*2CSA expression via chondroitin sulfate affinity purification (*var*2CSA positive line). Subsequently, half of the culture was treated with blasticidin (2.5 mM), which forced a switch to another *var* promoter (PF3D7_0223500) driving expression of the BSD resistance cassette^51^. Successful selection for parasites expressing *var2CSA* or PF3D7_0223500 was confirmed by RT-qPCR (Fig. S7). Subsequently, ChIP-qPCR was performed for the active histone modification H3K9ac, the inactive histone modification H3K9me3, the histone variant *Pf*H2A.Z and for *Pf*H3.3 for three different time points (15, 25 and 40 hpi). The switch from *var2CSA* expression to PF3D7_0223500 was confirmed at the epigenetic level: CSA-selected parasites showed high H3K9ac recoveries for the whole promoter region of the *var2CSA* gene and low H3K9me3 recoveries for the promoter as well as coding sequences (Fig. 4B). BSD-selected parasites, however, showed the opposite pattern (Fig. 4B). As also reported earlier, only the CSA-selected parasite line showed high occupancy of *Pf*H2A.Z close to the promoter start site 1500 bp upstream of the ATG^49^. Incorporation of *Pf*H3.3 into the promoter region of the *var2CSA* gene was clearly increased when the gene was active compared to its silenced state validating the observation made based on the ChIP-seq data. In summary, *Pf*H3.3 stably occupies the promoter region and coding sequence of the active *var* gene but is evidently less incorporated into the promoter and coding sequence of silenced *var* genes.

## Discussion

Here we present the first genome-wide analysis of histone variant H3.3 in the apicomplexan parasite, *P. falciparum*. Interestingly, *Pf*H3.3 has a distinct localization compared to other variants. While *Pf*H2A.Z and *Pf*H2B.Z^48^ as well as *Pf*CenH3^13^ appear to occupy AT-rich sequences, *Pf*H3.3 is most commonly found in GC-rich coding sequences and the subtelomeric repetitive regions (Fig. 1) and only temporarily occupies AT-richer intergenic regions (Fig. 3). In many animals, H3.3 is also found in coding regions, where it is incorporated during transcriptional elongation^24^,^25^. In *P. falciparum*, however, we could not observe a clear correlation between *Pf*H3.3 occupancy in euchromatic coding sequences and the steady-state mRNA level of the corresponding genes (Fig. 3, Fig. S6). Instead we find that most coding sequences are constantly marked by *Pf*H3.3 throughout the intraerythrocytic cycle and we observed a positive correlation between *Pf*H3.3 occupancy and the GC-content of the underlying DNA (Fig. 2A). This correlation is even more striking over exogenous DNA sequences that do not contain any *P. falciparum* “compatible” gene regulatory elements (Fig. 2B/C). Furthermore, although transcriptionally silent heterochromatic regions appear to have a generally lower *Pf*H3.3 occupancy, GC-richer coding sequences are yet more likely to contain some *Pf*H3.3 (Fig. 2A). These observations together suggest that *Pf*H3.3 incorporation to coding regions does not (necessarily) require transcriptional activity and *Pf*H3.3 occupancy is rather influenced by the GC-content of DNA sequence.

Next to constant occupancy of *Pf*H3.3 at euchromatic intergenic regions we observe clear *Pf*H3.3 marking at subtelomeric repeat sequences. In mammals, *Pf*H3.3 is also deposited at repressive chromatin regions and in mouse embryonic cells additionally at the telomeres^26^,^28^,^31^,^64^, suggesting that *Pf*H3.3 might serve a conserved telomere associated function. However, unlike in mouse embryonic stem cells^31^,^64^, *Pf*H3.3 occupancy seems to be uncoupled from K9me3 marking. In fact, *Pf*H3.3 appears to be specifically depleted from most heterochromatic regions - marked by K9me3 and HP1 in *P. falciparum* - except for the subtelomeric repetitive regions (Figs 1 and 2). Notably, these latter regions have the highest density of G quadruplex motifs in *P. falciparum* genome^65^. Since, earlier work in mouse established a potential link between G quadruplex sequences and H3.3 occupancy at the telomeres^66^, it is conceivable that a similar link might underlie the subtelomeric H3.3 localization in *P. falciparum*. However, it is even more straightforward to think these regions have high *Pf*H3.3 occupancy simply because they have a distinctly higher GC content (Fig. 2A), providing further support for the above model.

In addition to the GC-based correlation of *Pf* H3.3 localization to coding and subtelomeric sequences, we observe dynamic incorporation of *Pf*H3.3 into AT-rich intergenic regions, almost exclusively at the trophozoite stages. This stage is peculiar in two ways: i) this is the stage when extensive replication of the parasites genome begins; ii) this is the transcriptionally most active stage. Notably, in contrast to many other eukaryotic cells, cell division during the intraerythrocytic cycle of *P. falciparum* does not only double the DNA content, but rather results in 8 to 32 genome copies. Given that an enormous amount of histones needed to support the synthesis of the new chromatin it is conceivable that both H3 variants (*Pf*H3 and *Pf*H3.3) need to be incorporated during replication to the newly synthetized chromatin, after which *Pf*H3.3 might be preferentially retained at GC-rich sequences. A similar phenomenon is observed in mouse spermatocytes, when almost all histones are replaced by protamine to form a highly compact paternal chromatin, nucleosomes that are retained at unmethylated CpG-rich sequences, primarily contain H3.3^67^. Alternatively, the stage-specific incorporation of *Pf*H3.3 to promoter regions could be explained by temporal transcriptional activity, typical to intraerythrocytic stages of malaria parasites (Fig. 3). In fact in many other organisms H2A.Z and H3.3 containing nucleosomes are located around the transcriptional start site (for review see ref. 68). However, while our analysis revealed a moderate correlation between mRNA abundance and *Pf*H3.3 enrichment in promoter regions in particular at the trophozoite stage (Fig. S6) *Pf*H3.3 occupancy profiles in promoter regions are largely uncoupled from the dynamics of their transcriptional activity (Fig. 3). Accordingly, while we cannot completely exclude the possibility that *Pf*H3.3 is incorporated to some promoter regions in a transcription coupled manner, the extensive incorporation of *Pf*H3.3 to intergenic regions at the trophozoite stage is perhaps better explained by the replicative activity of this stage.

In mammalian cells, the different distribution patterns of H3.3 compared to H3 is achieved by histone- and site-specific chaperones. While H3.3 is deposited at euchromatic regions by the chaperone Histone regulator A (HIRA) replication-independently^20^,^69^, the death-associated protein (DAXX) together with the α-thalassemia and/or mental retardation X-linked syndrome protein (ATRX) mediates H3.3 deposition in pericentric heterochromatin and telomeric regions^26^,^27^. H3 deposition, however, is mediated by the chaperone CAF-1 replication-dependently^20^. Recognition of H3 and H3.3 by these chaperons is mediated by amino acid residues at positions such as 87 and 90^18^,^19^,^70^ which also differ in *Pf*H3 and *Pf*H3.3^18^,^19^,^42^,^50^,^70^ and might support differential recognition by different chaperon proteins. Thus far, however, only one *P. falciparum* protein with weak homology to both CAF1 and HIRA has been identified (PF3D7_0501800). Therefore, additional experiments will have to be performed to determine whether this protein supports the incorporation of both *Pf*H3 and *Pf*H3.3 during replication or yet unidentified *Pf*H3/*Pf*H3.3-specific chaperons exist that ensure differential genomic incorporation of *PfH3* and *Pf*H3.3.

Of particular interest is the specific *Pf*H3.3 incorporation to the promoter region of the active, but not inactive *var* gene (Fig. 4). This is similar to the earlier reported specific incorporation of the *Pf*H2A.Z/*Pf*H2B.Z double-variant nucleosomes around the transcription start site of the active *var* gene during the ring stage, when this gene is being actively transcribed^49^. Furthermore, the active *var* promoter is marked by two active marks (H3K9ac and H3K4me3), while the poised *var* gene carries H3K9ac and H3K4me2^58^,^71^,^72^. Interestingly, unlike *Pf*H2A.Z/*Pf*H2B.Z, *Pf*H3.3 is retained at this AT-rich *var* promoter even after active transcription has ceased (so called poised state). This raises the exciting possibility that *Pf*H3.3 may contribute to the epigenetic memory of *var* gene expression^57^,^58^, and enables the parasites to express the same *var* gene over several generations. In line with such hypothesis, nuclear transplantation experiments in *Xenopus laevis* showed that H3.3 mediates the epigenetic memory of certain genes; and H3.3 overexpression in nuclear transplant embryos even enhances this effect^73^,^74^,^75^. Furthermore, H3.3 enrichment at promoters has also been observed for inactive genes, which are potentially in a poised state^25^,^76^,^77^ as we observe it for the poised *var2CSA* gene during the second half of the intraerythrocytic cycle. All in all, these observations made in other organisms support the idea of a potential role of *Pf*H3.3 in epigenetic memory in *P. falciparum.* In order to investigate the functional relevance of *Pf*H3.3 in parasite biology in general or in *var* gene regulation in particular, we made several attempts to tag *Pf*H3.3 with a AID-GFP-glmS^78^,^79^ or GFP-glmS^79^ tag. Unfortunately, these attempts failed to result in viable parasite lines using these rather bulky conditional knock-down tags, hinting towards an essential function of *Pf*H3.3. Accordingly, whether *Pf*H3.3 is critical for “epigenetic memory” of *var* gene expression and how it could excerpt such function will be the subject of future studies.

In summary, we show that *Pf*H3.3 has a complex localization pattern that consists of conserved, but also parasite-specific features. It demarcates euchromatic coding and subtelomeric repetitive sequences in a GC-content coupled manner. Within the coding sequences it might promote the process of transcription by forming labile nucleosomes, as has been reported for other organisms. The strong marking of subtelomeric repetitive sequences resembles findings from higher eukaryotes, which could elude to a conserved, yet uncharacterized function. Excitingly, *Pf*H3.3 specifically marks the promoter region of the *var* gene in its active and poised, but not inactive state and could contribute to the process of antigenic variation which is critical to immune evasion of this deadly pathogen.

## Methods

### Generation of Ty1-tagged *Pf*H3.3 parasites

In order to profile *Pf*H3.3, 3D7 *P. falciparum* parasites were transfected with a pARL-1a- plasmid^80^ encoding a N-terminally Ty1-tagged version of *Pf*H3.3 (PF3D7_0617900) under the control of the chloroquine resistance transporter promoter (similar to pARL-1a-Ty1-H2A in ref. 47) and cultured in presence of 40 nM WR99210. To generate *Pf*H3.3 profiles from endogenously expressed *Pf*H3.3, NF54-DCJ *P. falciparum* parasites^51^ were transfected with a pHH1-plasmid^81^ encoding a C-terminally Ty1-tagged version of *Pf*H3.3 (PF3D7_0617900) and a CTG instead of an ATG as “start codon” (Fig. S1A). Parasites were cultured in presence of 2.5 nM WR99210. Single crossover integration of the plasmid resulted in parasites that express a fully functional *Pf*H3.3-Ty1 under the endogenous promoter (ATG as start codon) and a non-functional untagged *Pf*H3.3 (CTG as start codon) (Fig. S1B). A clonal parasite line was obtained by limiting dilution. Correct integration of the pHH1-plasmid and absence of wild type parasites was confirmed by performing Southern Blot analysis (Fig. S1B,C). Expression of episomal and endogenously-expressed tagged *Pf*H3.3 was confirmed using the monoclonal anti-Ty1 antibody (BB2)^82^ that exclusively recognizes the tagged *Pf*H3.3 protein on Western blot (Fig. S1D).

### Parasite culture and collection of parasites

Parasites were cultured in standard RPMI medium supplemented with 10% human serum (AB), 0.2% NaHCO3 and 2.5% human O^+^ red blood cells. Culturing occurred in 250 ml tissue culture flasks placed in candle jars or in an incubator with a gas composition of 3% O_2_, 4% CO_2_ and 93% N_2_ and incubated at 37 °C. Parasites were synchronized with sorbitol treatments and Percoll gradient centrifugations as described previously^47^. During collections, medium was changed every 10 hours, but not less than 10 hours before collection to ensure optimal parasite growth. After 20 hours post invasion (hpi), medium volume was doubled. During each medium change parasite cultures were mixed to minimize growth differences within single culture flasks. For the generation of genome-wide *Pf*H3.3 profiles early ring (10 hpi), late ring (20 hpi), trophozoite (30 hpi) and schizont stages (40 hpi) were collected. For the ChIP-qPCR investigation of *Pf*H3.3 *var*2CSA- or PF3D7_0223500-selected parasites, ring, trophozoite and schizont stages were collected at 15, 25 and 40 hpi. Parasite cultures used for RNA extraction or native ChIP were immediately placed and processed on ice (4 °C). Parasite cultures used for cross-linked ChIP were immediately treated with 1% formaldehyde (final concentration) and incubated at 37 °C for 10 min while shaking. Cross-linking reaction was quenched by adding 0.125 M glycine (final concentration). All subsequent steps were performed at 4 °C unless noted otherwise. To remove contaminating human white blood cells that would cause considerable background upon sequencing, all blood used for collection of sequence samples was filtered through Plasmodipure filters (EuroProxima).

#### Etic statement

Blood and serum was obtained from the local blood bank (Sanquin) with the full consent of healthy donors to use this material for malaria research.

### *var*2CSA (PF3D7_1200600) and PF3D7_0223500 selection

Endogenously *Pf*H3.3-Ty1 expressing NF54-DCJ *P. falciparum* parasites were selected for *var*2CSA expression by affinity purification as in ref. 83. For this purpose, petri dishes (150 × 15 mm, BD biosciences Falcon 351058) were coated with Chondroitin sulfate A (0.05% CSA in PBS) overnight and subsequently blocked with 1% Casein/PBS solution for at least one hour and rinsed with RPMI twice. In the meanwhile parasite cultures were centrifuged, resuspended in RPMI containing 10% human serum and transferred to the petri dishes. Subsequently, petri dishes containing the parasite culture were incubated for 30 min at 37 °C in a candle jar. Afterwards, unbound parasites and non-infected erythrocytes were removed by gentle RPMI washes. Bound parasites were extensively resuspended in complete medium, fresh blood was added, and parasites were cultured as described above. *var*2CSA panning was repeated several times to achieve an increasing proportion of *var*2CSA-expressing parasites. Subsequent switching to PF3D7_0223500 expression, was enforced by culturing the parasites with 2.5 mM Blasticidin^51^. Successful *var* gene selection was confirmed via RNA extraction (RNeasy mini kit, Qiagen) followed by cDNA synthesis and subsequent quantitative real time PCR (qPCR) using *var* gene specific primers^71^,^84^ (Fig. S7A,B).

### cDNA synthesis and qPCR

To synthesize the cDNA 0.5–5 ug of total RNA were mixed with random hexamer primers (0.5 ug, Roche), OligodT12-18 (0.5 ug, Invitrogen) and dNTPs (0.5 mM in the final volume of 20 ul, Invitrogen) and incubated for 5 min at 70 °C. Then first strand synthesis was performed for 1 hour at 42 °C in First Strand Buffer supplemented with DTT (10 mM), Superscript III (200 units) and RNasin Plus RNase inhibitor (40 units, Promega), after which superscript III was inactivated by incubation at 70 °C for 15 minutes. RT “minus” reactions (where Superscript III was replaced by water) were performed in all cases as negative control. Subsequent qPCR was performed using the CFX96 Real Time Systems C1000 Touch Thermal Cycler. *P. falciparum* genomic DNA served as standard (500 pg, 50 pg, 5 pg). SYBRgreen supermix (BioRad) and primers were mixed according to manufacturer’s instructions. qPCR was performed in 96 well plate format (BioRad) using the following protocol: 95 °C for 3 min, (94 °C for 10 sec, 52 °C for 30 sec, 68 °C for 30 sec) 39x, 95 °C 1 min, 65 °C 1 min and a gradient from 65 °C to 94.5 °C with a 0.5 °C increase every 10 sec.

### Western Blot analysis

Nuclear extracts from the 3D7 mother line (~40 ug), the 3D7 parasites expressing Ty1-*Pf*H3.3 episomally (~60 ug) and the NF54-DCJ parasites expressing *Pf*H3.3-Ty1 under the endogenous promoter respectively, were separated on 18% SDS-PAGE and transferred to a protean nitrocellulose membrane (Whatman, 0.45 mm). The membrane was probed with mouse anti-Ty1 antibody (1:2000, BB2 antibody in ref. 82) and rabbit anti-histone H3 antibody (1:3000, Abcam ab1791) and secondary goat anti-mouse IRDye680 (1:10000, LI-COR Biosciences 926-32220) and goat anti-rabbit IRDye 800CW (1:10000, LI-COR 926-32211). Fluorescence was measured on the Odyssey system (LI-COR Biosciences).

### Southern Blot analysis

Genomic DNA was isolated from native nuclei using phenol-chloroform extraction as described previously^47^. 2.5 μg of genomic DNA and 0.5 ng of plasmid DNA were digested with EcoRI and XbaI (NEB) (Fig. S1B). A *Pf*H3.3 sequence specific hybridization probe was generated by PCR labelling using Digoxigenin-11-dUTP (DIG) (alkali-labile, Roche) according to manufacturer’s instructions. The PCR reaction was performed using the Advantage^®^ cDNA PCR Kit and Polymerase Mix (Clonetech) and a DIG-dUTP to dTTP ratio of 1:3 under the following PCR conditions: 5 min 95 °C; 30 s 95 °C, 30 s 52 °C, 90 s 68 °C (35 cycles); 5 min 68 °C (Fig. S1B). Gel preparation, subsequent DNA transfer to a positively charged Nylon membrane (Hybond-N^+^, GE Healthcare Amersham), prehybridization (DIG Easy Hyb Kit from Roche, 65 °C) and probe-DNA hybridization (DIG Easy Hyb Kit from Roche, 65 °C) were all performed according to manufacturer’s recommendations. The detection of the hybridized probe was performed using the DIG Wash and Block Buffer Set, the anti-Digoxigenin antibody (anti-Digoxigenin-AB conjugate Fab frag; 1:10 000 dilution) and the CDP-Star Kit (ready-to-use) from Roche according to manufacturer’s instructions.

### Chromatin immunoprecipitation

After erythrocyte and parasite lysis using 0.05% saponin and a hypotonic buffer (10 mM Tris pH 8.0, 3 mM MgCl_2_, 0.2% NP-40, Roche Protease Inhibitor Cocktail) respectively, native nuclei (ChIP-seq of episomal Ty1-*Pf*H3.3 and *var* gene switch experiment, see below) or formaldehyde-cross-linked nuclei (ChIP-seq experiment) were separated from cell debris using a 0.25 M sucrose buffer cushion as described before^47^. Subsequent, cross-linked chromatin was prepared as described in ref. 85 with a few adjustments. In short, for generation of genome-wide *Pf*H3.3 profiles, enzymatic MNase digestion of cross-linked chromatin was performed using digestion buffer (50 mM Tris pH7.4, 4 mM MgCl2, 1 mM CaCl2, 0.075% NP40, Roche Protease Inhibitor Cocktail) with 0.5 U MNase (Worthington Biochemicals Corporation) in 150 μl aliquots for 6 min (10 hpi), 12 min (20 hpi) or 13 min (30 hpi & 40 hpi) at 37 °C. Optimal digestion times were empirically determined for each stage by test-digestion of a nuclear aliquot for varying times, to obtain primarily mono-nucleosomal DNA fragments. Digestion reactions were stopped by adding 150 μl quenching solution (2% Triton X100, 0.6% SDS, 300 mM NaCl, 6 mM EDTA, Roche Protease Inhibitor Cocktail) and placing the samples on ice. Subsequently, digested chromatin was sonicated for 6 × 10 sec (setting low, BioruptorTM Next Gen, Diagenode) to free cross-linked chromatin from nuclear membranes. Approximately 200 ng of digested DNA-containing cross-linked chromatin was incubated in ChIP buffer (150 mM NaCl, 20 mM Tris pH 8.0, 2 mM EDTA, 1% Triton X-100, 0.15% SDS, Roche Protease Inhibitor Cocktail) with 2 μg of anti-Ty1, 1 ug of anti-histone H3 (recognizes *Pf*H3 and *Pf*H3.3, Abcam Ab1791) or 1 μg polyclonal anti-IgG (Upstate 12-370) overnight at 4 °C while rotating, followed by the addition of 10 μl ProtA and 10 ul ProtG dynabeads (Life Technologies, 10008D and 10009D) and further incubation at 4 °C for 2 h. For the H3core antibody (capturing histone *Pf*H3 and *Pf*H3.3) one ChIP reaction was performed, for *Pf*H3.3 (anti-Ty1) four ChIP reactions were performed in parallel per life cycle stage to ensure sufficient amount of DNA for subsequent ChIP-seq. After washing with three different buffers (buffer1, 1x: 20 mM Tris pH 8.0, 2 mM EDTA, 1% Triton-X100, 0.1% SDS, 150 mM NaCl; buffer 2, 2x: 20 mM Tris pH 8.0, 2 mM EDTA, 1% Triton-X100, 0.1% SDS, 500 mM NaCl and buffer 3, 2x: 10 mM Tris pH 8.0, 1 mM EDTA) immunoprecipitated chromatin was eluted using 1% SDS and 0.1 M NaHCO_3_. Immunoprecipitated chromatin was decross-linked (1% SDS, 0.1 M NaHCO_3_, 1 M NaCl) overnight at 45 °C shaking, followed by DNA isolation via QIAquick column purification (Qiagen).

To investigate *Pf*H3.3 and *Pf*H2A.Z occupancy as well as H3K9ac and H3K9me3 marking of the *var*2CSA locus of *var*2CSA- or PF3D7_0223500-selected parasites, chromatin was extracted and native ChIPs were performed as described previously^47^. Native chromatin was digested with 0.5 U MNase in 150 μl aliquots for 4 min (15 hpi *var*2CSA-selected parasites), 7 min (15 hpi, 25 hpi, 40 hpi PF3D7_0223500-selected parasites and 25 hpi *var*2CSA-selected parasites) and 10 min (40 hpi Var2CSA-selected parasites) at 37 °C in a waterbath and regular tapping of the tubes (again optimal digestion times to primarily mono- nucleosomal fragments were determined empirically by a test-digestion of an nuclear-aliquot from each sample). Subsequent chromatin extraction was performed in low-salt buffer (buffer1: 50 mM Tris pH 7.4, 4 mM MgCl_2_, 1 mM CaCl_2_, Roche Protease Inhibitor Cocktail; buffer2: 10 mM Tris pH 7.4, 1 mM EDTA, Roche Protease Inhibitor Cocktail; buffer3: 1 mM Tris pH 7.4, 0.2 mM EDTA, Roche Protease Inhibitor Cocktail) and incubated with 1 volume ChIP incubation buffer (100 mM NaCl, 19.7 mM Tris pH 7.4, 6.25 mM EDTA, 1% Triton X-100, 0.1% SDS). For native ChIP reactions, native chromatin (containing about 200 ng DNA) from each time point of *var*2CSA- and PF3D7_0223500-selected parasites was incubated with 2 μg of anti-Ty1 (*Pf*H3.3), 0.5 μl polyclonal H2A.Z^47^, 1 μg of polyclonal anti-H3K9/14ac antibody (Diagenode, C15410200), 1 μg of polyclonal H3K9me3 (in house, corresponds to Merck Millipore 07-442) or 1 μg polyclonal IgG (Upstate 12-370) overnight at 4 °C followed by the addition of 20 μl ProtA/G dynabeads and further incubation at 4 °C for 2 h. 200 ng of native chromatin were kept as input. After washing with buffers three different buffers (buffer 1, 1x: 50 mM Tris pH 7.5, 10 mM EDTA, 100 mM NaCl, 0.5% Triton X-100, 0.05% SDS; buffer 2, 2x: 50 mM Tris pH 7.5, 10 mM EDTA, 150 mM NaCl, 0.5% Triton X-100, 0.05% SDS and buffer 3, 2x: 50 mM Tris pH 7.5; 10 mM EDTA, 250 mM NaCl) immunoprecipitated DNA was eluted and purified using PCR purification columns.

### ChIP-qPCR

To test relative enrichment of *Pf*H3.3-Ty1, H2A.Z, H3K9ac and H3K9me3 xlChIP was performed as described above. qPCR (Bio-Rad MyIQ) on each ChIP sample was performed alongside with a 1:10 dilution series of the corresponding input DNA to calculate recovery as a percentage of input. Recovery values for the var2CSA locus where normalized across the different samples using primer sets for regions that are invariably marked with the histone mark/variant. For H3K9ac, PfH2A.Z and PfH3.3 primers for actin (PF3D7_1246200, coding sequence and -1100bp promoter region) and seryl-tRNA synthetase (PF3D7_0717700, coding sequence), HSP70 (PF3D7_0818900, coding sequence and -1000 promoter regions) and 18s RNA (PF3D7_1148600, coding sequence) were chosen. For H3K9me3 primers for bp the telomere repetitive regions, a rifin (PF3D7_0324500, - 400bp promoter regions) and AP2G (PF3D7_1222600, coding sequence). Normalization factor was calculated by dividing the average of the recoveries from the control primers within each sample with the average of the average recovery values across the samples. Finally, all recovery values from the var2CSA specific primers have been multiplied by the normalization factors. The normalization factor scaled between 0.75 and 1.8.

### ChIP-seq library preparation

For each sequencing library generated from endogenously *Pf*H3.3-Ty1 expressing NF54-DCJ *P. falciparum* parasites 3 ng of ChIP DNA were end repaired, extended with 3′ A-overhangs, and ligated to barcoded NextFlex adapters (Bio Scientific)^86^. Subsequent library amplification was performed under similar conditions as described previously^85^ with a few adjustments: First, 2x KAPA HiFi HotStart ready-mix (KAPA Biosystems) and NextFlex primers were used for 4 cycles of PCR amplification (98 °C for 2 min; 4 cycles of 98 °C for 20 sec, 62 °C for 3 min; 62 °C for 5 min). Subsequently, amplified libraries were size selected for 270 bp (mono-nucleosomes + ~125 bp NextFlex adapter) using 2% E-Gel Size Select Agarose Gels (Thermo Fisher Scientific) and again amplified as described above for 9 cycles. To deplete adapter dimers and clean-up the DNA, libraries were subsequently purified with Agencourt AMPure XP bead (Beckman Coulter) (1:1 library beads ratio) before sequencing. Sequencing libraries generated from episomally Ty1-*Pf*H3.3-expressing parasites were generated as described in refs 47,86.

### RNA-seq library preparation

The preparation of strand-specific RNA-seq libraries was performed as in ref. 85. In short, after total RNA isolation (with on-column DNase treatment) and oligo-dT-selection^87^ PolyA-selected RNA was further treated with DNase (TURBO DNase, Ambion) to remove remaining genomic DNA. Subsequently, 1.2 μg PolyA-selected total RNA equivalent (see ref. 87 for details) for 10 hpi and 2 μg for 20 hpi, 30 hpi and 40 hpi were fragmented by means of alkaline hydrolysis (5x fragmentation buffer: 200 mM Tris acetate pH 8.2, 500 mM potassium acetate, 150 mM magnesium acetate) for 2 min at 85 °C in 250 μl volume. RNA was precipitated overnight and checked for genomic DNA contamination using qPCR, followed by - if needed - another DNase treatment and RNA precipitation for clean-up. Remaining material was processed for strand-specific RNA-seq as in ref. 85. To prevent unwanted DNA-dependent second strand cDNA synthesis - a major source of artificial antisense ‘transcripts’^88^ −0.2 μg Actinomycin D was included into the first strand synthesis reaction, which was followed by a 15 min 70 °C enzyme deactivation step and QIAquick MinElute purification (Qiagen). During second strand synthesis replacement of dTTP with dUTPs resulted in incorporation of U-bases into the second cDNA strand. Subsequently, 2 ng double stranded cDNA were used for each sequencing library which were prepared as described above with the exception that after adaptor ligation samples were treated with USER enzyme (New England Biolabs) for 15 min at 37 °C that resulted in dUTP-dependent second strand-specific degradation, followed by a first amplification cycle (4 cycles), size-selection for 300–400 bp fragments, a second amplification cycle (9 cycles) and Ampure Beads purification (see above).

### High throughput sequencing

ChIP-seq and strand-specific RNA-seq libraries generated from endogenously *Pf*H3.3-Ty1 expressing NF54-DCJ parasites were sequenced on a HiSeq 2000 system (Illumina) to obtain 100 bp single-end reads (TruSeq SE Cluster Kit v2). ChIP-seq libraries generated from episomal Ty1-*Pf*H3.3 expressing parasites were sequenced on a GAIIx system (Illumina) to obtain 75 bp single-end reads (Standard Cluster Generation KitV4). 100 bp and 75 bp reads respectively were mapped against the *Plasmodium falciparum* genome assembly (PlasmoDB v6.1) for ChIP samples or against the *Plasmodium falciparum* annotated transcripts (PlasmoDB v6.1) for mRNA samples using BWA^89^. Single-end ChIP and RNA reads were filtered to mapping quality ≥15 and only uniquely mapped reads (between 13.5 and 23.5 million reads for ChIP-seq and 15.2 and 26.8 million reads for RNA-seq) were used for further analysis.

### High-throughput sequencing data analysis

To visualize ChIP-seq and RNA-seq data in the UCSC browser, all libraries were normalized to the amount of mapped reads per million (RPM). For log2 ratio tracks *Pf*H3.3 values were divided through the respective *Pf*H3core values and log2 was calculated (“unmappable” regions containing the value 0 were discarded). Bedgraph.gz files were generated using bedtools v2.20.1. Genome browser tracks were set to ‘mean’ as windowing function and ‘16’ as smoothing window function.

To investigate the correlation of *Pf*H3.3 with the GC-content of the *Plasmodium falciparum* genome the genome was binned into 150 bp windows and the GC-content as well as *Pf*H3.3 and *Pf*H3core tags were calculated in each window. *Pf*H3.3 tags were divided through *Pf*H3core tags (windows containing the value 0 were discarded), log2 transformed and plotted over the GC-content as a density plot using the python package seaborn 0.6.0 in ipython notebook (Enthought Canopy 1.5.5.3123). For more detailed analysis the 150 bp windows were intersected with different chromatin domains e.g. heterochromatic or euchromatic regions (at least 50% overlap) using bedtools v2.20.1 and depicted as described above.

In order to investigate the correlation of *Pf*H3.3 in upstream regions, downstream regions or coding sequences with mRNA abundance, the genome was subdivided as described^85^. In short, intergenic regions between convergent and divergent genes were split in half and each half was assigned to the nearest gene. Intergenic regions between genes with the same orientation were split in 3/4 and 1/4. The longer part was assigned to the upstream region of the nearest gene and the shorter part to the downstream region of the other gene. All upstream and downstream regions were shortened to a maximum of 3 kb as this covers the vast majority of upstream regions in full length, but excludes some extremely long intergenic regions. *Pf*H3.3 and *Pf*H3core tags were calculated within each upstream region, downstream region and coding sequence, normalized to the amount of reads per kb per million mapped reads (RPKM) and *Pf*H3.3/*Pf*H3core ratios (*Pf*H3.3 occupancy) were determined. To assess RNA abundance of each gene, RNA-seq data were mapped against the annotated *P. falciparum* transcriptome, tags were counted for each transcript (excluding tRNAs, rRNAs, mitchochondrial and apicoplast RNA) and RPKM values were calculated. To illustrate the dynamics of RNA expression during intraerythrocytic development, the relative RNA abundance was calculated by dividing the amount of tags per transcript of each intraerythrocytic stage through the sum of tags per transcript of all four investigated intraerythrocytic stages (Heterochromatic genes and 25% of euchromatic genes with the lowest expression has been excluded from this analysis). Subsequently, RNAs were clustered into eight groups according to their expression pattern throughout the intraerythrocytic cycle using k-means clustering (Multi Experiment Viewer 4.9.0). Clusters were visualized in a heatmap. After calculating the relative *Pf*H3.3 occupancy in upstream regions for all four intraerythrocytic stages as described above, the profile of *Pf*H3.3 occupancy in upstream regions was matched to the respective RNA expression profile. The same method was applied to coding sequences and downstream regions.

To illustrate the relationship between *Pf*H3.3 enrichment and gene expression level, RPKM values were log10 transformed and plotted against log2 transformed *Pf*H3.3 occupancy levels of matching upstream region, downstream region and coding sequence.

### Data Availability

ChIP-seq and RNA-seq datasets will be available via GEO (accession number GSE80466) and PlasmoDB.

## Additional Information

**How to cite this article**: Fraschka, S. A.-K. *et al*. H3.3 demarcates GC-rich coding and subtelomeric regions and serves as potential memory mark for virulence gene expression in *Plasmodium falciparum*. *Sci. Rep.* **6**, 31965; doi: 10.1038/srep31965 (2016).

## Supplementary Material

Supplementary Information
