# Supplementary material for: H3.3 demarcates GC-rich coding and subtelomeric regions and serves as potential memory mark for virulence gene expression in Plasmodium falciparum
Source: Sci Rep. 2016 Aug 24;6:31965. doi: 10.1038/srep31965 (PMC4995406; doi:10.1038/srep31965)

SUPPLEMENTARY INFORMATION

H3.3 demarcates GC-rich coding and subtelomeric regions and serves as potential memory mark for virulence gene expression in *Plasmodium falciparum*

Sabine Anne-Kristin Fraschka [1], Rob Wilhelmus Maria Henderson [1, #], and Richárd Bártfai [1,*]

[1] Department of Molecular Biology, Radboud University, Nijmegen, the Netherlands

* Corresponding author; r.bartfai@science.ru.nl

[#] Current address: TropIQ Health Sciences, Nijmegen, the Netherlands

Figure S1: Generation of endogenously tagged *Pf*H3.3-Ty1 parasite line.

(A) Map of the pHH1-*Pf*H3.3-Ty1 integration vector. (B) Schematic representation of the fragments obtained from the i) integration vector ii) the wild type and iii) tagged *Pf*H3.3 locus after EcoRI/XbaI digestion. Gel picture shows the successful DIG labelling of 349bp DNA probe corresponding to the coding sequence of *Pf*H3.3. (C) Southern Blot confirming successful integration of *Pf*H3.3-Ty1 into the parasite's genome of two independent clones of the *P. falciparum* NF54- DCJ line. (D) Western Blot demonstrating the expression of Ty1-tagged *Pf*H3.3 both from an episomal construct as well as from the endogenous locus. The blot was stained with Ty1-tag specific antibody (BB2, red) and H3core antibody (green).

Figure S2: Wild type and endogenously tagged *Pf*H3.3-Ty1 parasites show a similar RNA expression pattern.

RPKM values were calculated for wild type 3D7 [85] and endogenously tagged *Pf*H3.3-Ty1 DCJ parasites for all transcripts excluding tRNAs, rRNAs, mitchochondrial and apicoplast RNA. Log10 transformed RPKM values are depicted for early rings, late rings, trophozoites and schizonts. Pearson correlation values are displayed at the lower right corner of the graphs. A red square represents *Pf*H3.3.

Figure S3: Ratio tracks of *Pf*H3.3-Ty1 ChIP-seq obtained from two different parasite lines at two stages of intraerythrocytic development.

(A) ChIP-seq ratio tracks of trophozoite (T) and schizont (S) stages are depicted in purple and violet for two biological replicates of the episomally Ty1-*Pf*H3.3 expressing 3D7 parasite line (N-terminally tagged *Pf*H3.3, native ChIP, linear amplification protocol, normalized over input) and in orange for endogenously *Pf*H3.3-Ty1 expressing NF54-DCJ parasites (C-terminally tagged *Pf*H3.3, cross-linked ChIP, KAPA amplification protocol, normalized over H3core values).

(B) Pearson correlation values calculated from log2 transformed *Pf*H3.3/Input or *Pf*H3.3/H3core values for all three ChIPs-seq data sets in two intraerythrocytic stages.

Figure S4: Centromere regions are *Pf*H3.3 depleted and marked by *Pf*CenH3

*Pf*CenH3, but also *Pf*H3.3-Ty1 and H3core ChIP-seq tracks were normalized over input since the H3core antibody only captures *Pf*H3 and *Pf*H3.3. *Pf*CenH3 clearly marks centromeres (upper panel) whereas *Pf*H3.3 (middle panel) but also *Pf*H3 (lower panel) is depleted from these sites. Normalized ChIP-seq data are depicted from schizont stages (40hpi). Centromeres are indicated by a black boxes.

Figure S5: *Pf*H3.3 localizes to GC-rich regions within the *P. falciparum* genome throughout intraerythrocytic development.

Density plots depicting *Pf*H3.3 levels in relation to GC-content. *Pf*H3.3 levels and GC-content were calculated genome-wide per 150 bp windows.
(A) Data depicted are generated from endogenously tagged *Pf*H3.3-Ty1 DCJ parasites for four different intraerythrocytic stages: Early rings (10hpi), late rings (20hpi), trophozoites (30hpi) and schizonts (40hpi).
(B) Data depicted are generated from episomally Ty1-*Pf*H3.3 expressing parasites (see Fig. S3 "episomal 1") at schizont stage.
IG: intergenic region, CS: coding sequence, Sub. Rep.: subtelomeric repetitive region. Pearson correlation values are displayed at the lower right corner of the graphs.

Figure S6: Correlation between steady state mRNA abundance and *Pf*H3.3 occupancy at four intraerythrocytic stages.
Scatter plots depicting log10 transformed RPKM values from all transcripts excluding tRNAs, rRNAs, mitchochondrial and apicoplast RNA in relation to log2 transformed *Pf*H3.3/H3core ratios in upstream regions, coding sequences and downstream regions. RNA- and ChIP-seq analysis were performed on early rings, late rings, trophozoites and schizonts from the endogenously tagged *Pf*H3.3-Ty1 DCJ parasite line. Pearson correlation values are displayed at the higher right corner of the graphs.

Figure S7: Endogenously *Pf*H3.3-Ty1 expressing NF54-DCJ *P. falciparum* parasites were successfully selected for *var*2CSA and PF3D7_223500 expression .
(A) Two independent, endogenously *Pf*H3.3-Ty1 expressing NF54-DCJ *P. falciparum* parasites clones were selected for *var*2CSA expression by repeated rounds of *var*2CSA panning. Successful *var* gene selection was confirmed via RNA extraction followed by cDNA synthesis and subsequent qPCR using one *var*2CSA specific primer pair and 10 primers pairs specific for 10 other *var* genes.
(B) CSA-selected parasites were treated with Blasticidin. This selected for parasites expressing PF3D7_223500 since in this parasite line the PF3D7_223500 promoter drives the expression of the BSD resistance cassette (*bsd*). Successful *var* gene switch was confirmed via RNA extraction followed by cDNA synthesis and subsequent qPCR using one specific primer pair for *bsd*, one specific primer pair for *var2*CSA and primers pairs specific for 8 other *var* genes.

Fig. S1

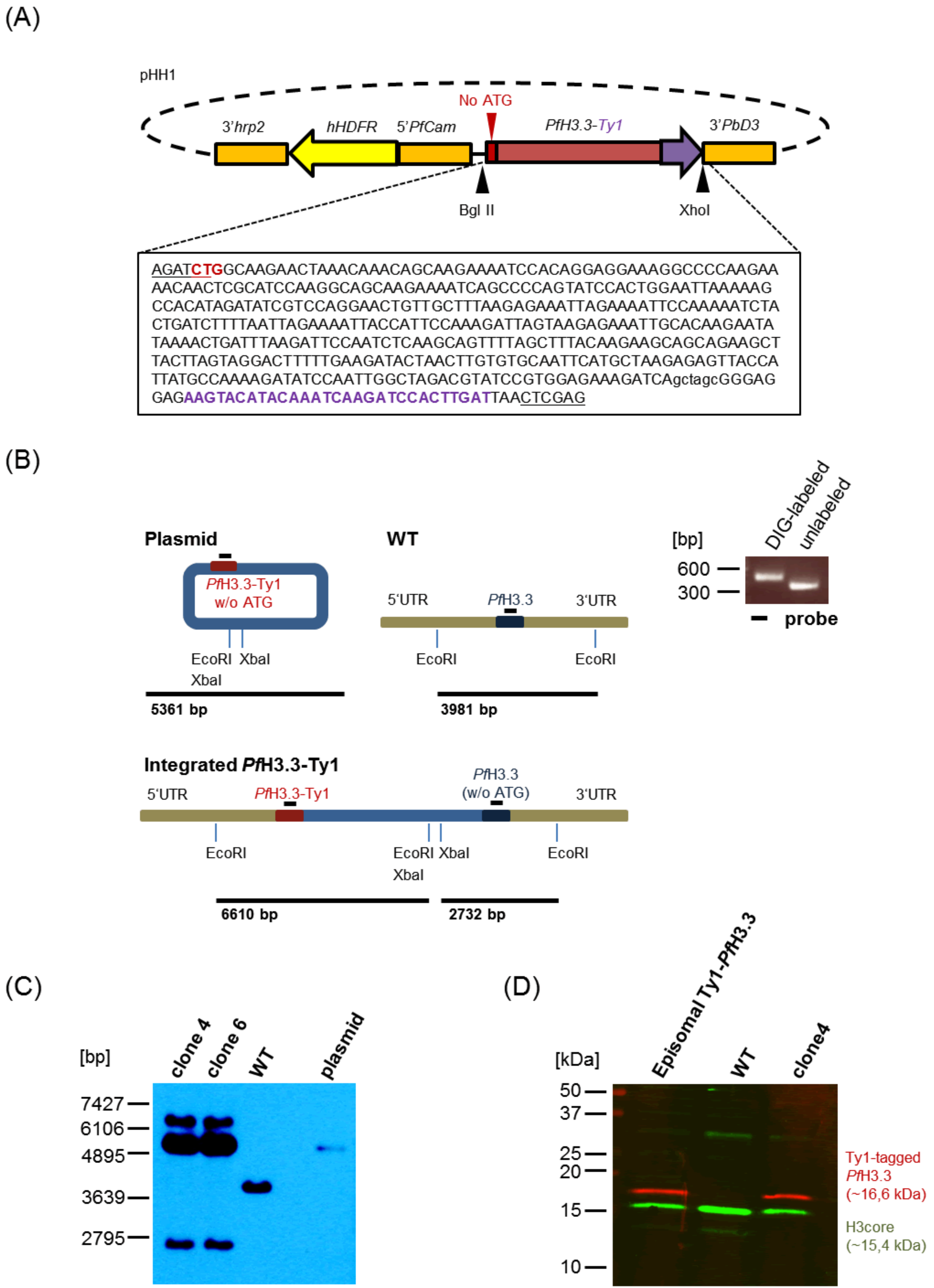

(A)
pHH1
No ATG
3'hrp2
hHDFR
5'PfCam
PfH3.3-Ty1
3'PbD3
Bgl II
XhoI
AGATCTGGCAAGAACTAAACAAACAGCAAGAAAATCCACAGGAGGAAAGGCCCCAAGAA
AACAACTCGCATCCAAGGCAGCAAGAAAATCAGCCCCAGTATCCACTGGAATTAAAAAG
CCACATAGATATCGTCCAGGAACTGTTGCTTTAAGAGAAATTAGAAAATTCCAAAAATCTA
CTGATCTTTTAATTAGAAAATTACCATTCCAAAGATTAGTAAGAGAAATTGCACAAGAATA
TAAAACTGATTTAAGATTCCAATCTCAAGCAGTTTTAGCTTTACAAGAAGCAGCAGAAGCT
TACTTAGTAGGACTTTTTGAAGATACTAACTTGTGTGCAATTCATGCTAAGAGAGTTACCA
TTATGCCAAAAGATATCCAATTGGCTAGACGTATCCGTGGAGAAAGATCAgctagcGGGAG
GAGAAGTACATACAAATCAAGATCCACTTGATTAACTCGAG
(B)
Plasmid
PfH3.3-Ty1
w/o ATG
EcoRI Xbal
Xbal
5361 bp
WT
5'UTR
PfH3.3
3'UTR
EcoRI
EcoRI
3981 bp
[bp]
DIG-labeled
unlabeled
600
300
probe
Integrated PfH3.3-Ty1
5'UTR
PfH3.3-Ty1
PfH3.3
(w/o ATG)
3'UTR
EcoRI
EcoRI Xbal
Xbal
EcoRI
6610 bp
2732 bp
(C)
[bp]
clone 4
clone 6
WT
plasmid
7427
6106
4895
3639
2795
(D)
[kDa]
Episomal Ty1-PfH3.3
WT
clone4
50
37
25
20
15
10
Ty1-tagged PfH3.3 (~16,6 kDa)
H3core (~15,4 kDa)

Fig. S2

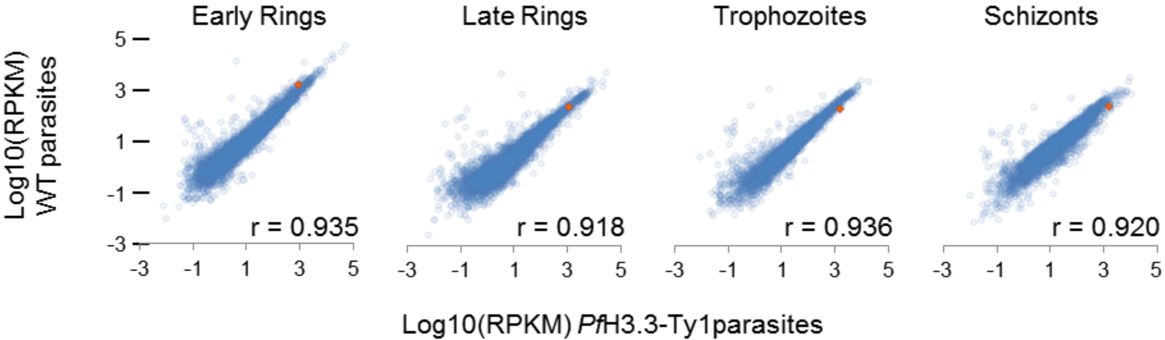

Early Rings
Late Rings
Trophozoites
Schizonts
Log10(RPKM) WT parasites
5
3
1
-1
-3
r = 0.935
r = 0.918
r = 0.936
r = 0.920
-3 -1 1 3 5
Log10(RPKM) *Pf*H3.3-Ty1parasites

Fig. S3

(A)

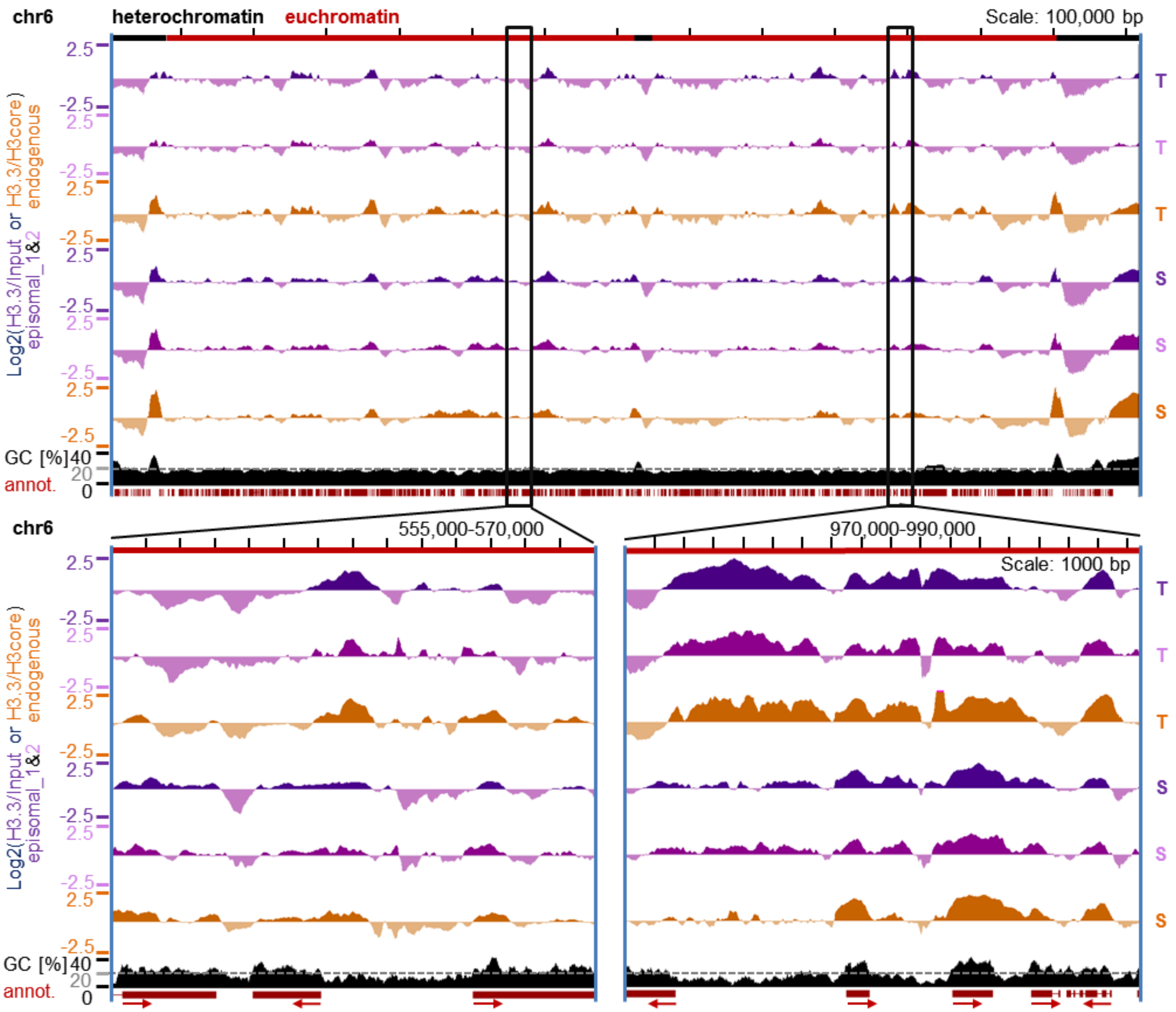

(B)

Pearson correlation of log2(H3.3/Input or H3.3/H3core) values

Tropozoites		Schizonts	
episomal 1 – episomal 2	0.843	episomal 1 – episomal 2	0.840
episomal 1 – endogenous	0.864	episomal 1 – endogenous	0.786
episomal 2 – endogenous	0.760	episomal 2 – endogenous	0.710

Fig. S4

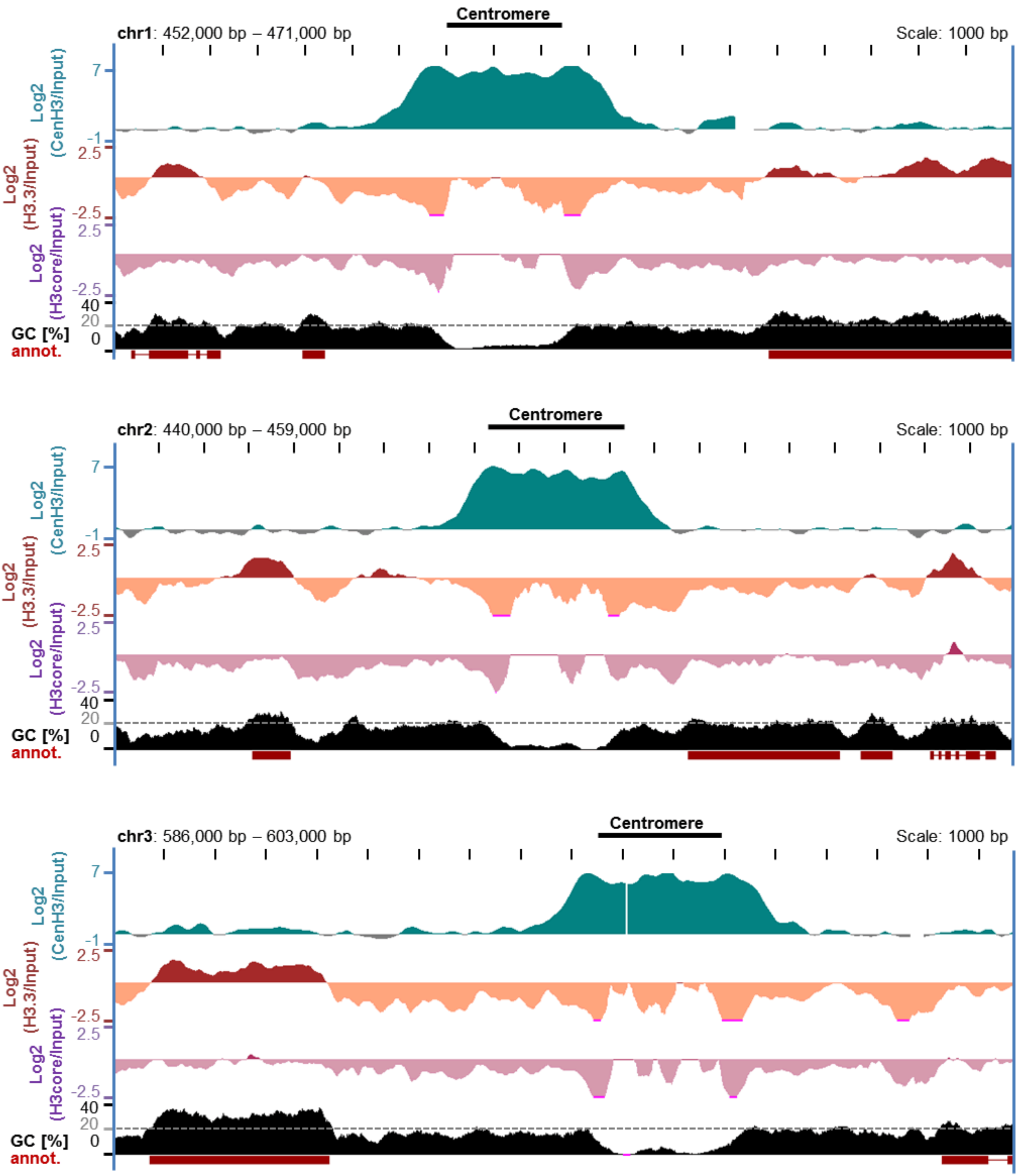
Centromere
chr1: 452,000 bp – 471,000 bp
Scale: 1000 bp
Log2 (CenH3/Input)
7
-1
Log2 (H3.3/Input)
2.5
-2.5
Log2 (H3core/Input)
2.5
-2.5
GC [%]
40
20
0
annot.
Centromere
chr2: 440,000 bp – 459,000 bp
Scale: 1000 bp
Log2 (CenH3/Input)
7
-1
Log2 (H3.3/Input)
2.5
-2.5
Log2 (H3core/Input)
2.5
-2.5
GC [%]
40
20
0
annot.
Centromere
chr3: 586,000 bp – 603,000 bp
Scale: 1000 bp
Log2 (CenH3/Input)
7
-1
Log2 (H3.3/Input)
2.5
-2.5
Log2 (H3core/Input)
2.5
-2.5
GC [%]
40
20
0
annot.

Fig. S5

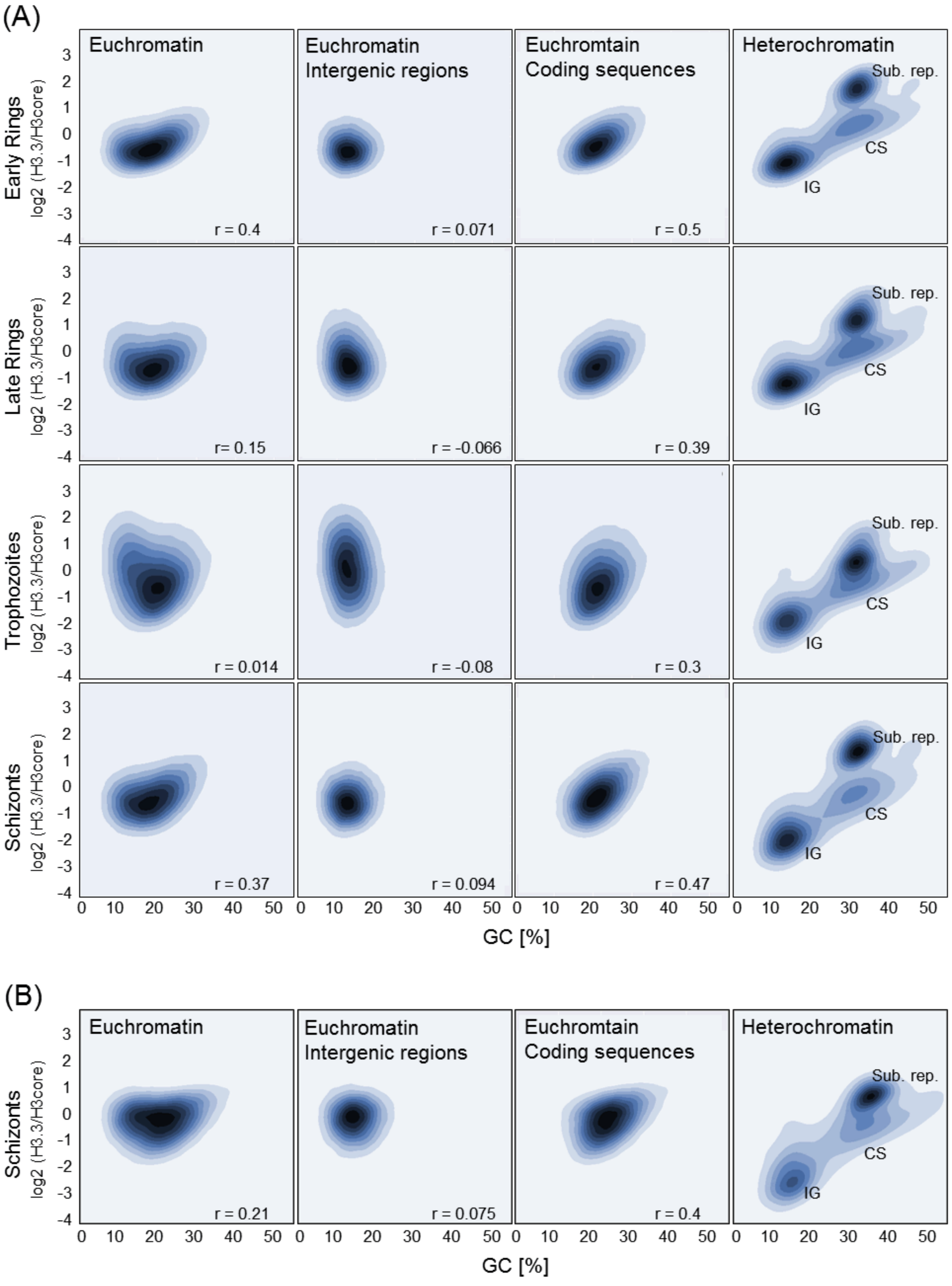

(A)
Euchromatin
Euchromatin
Intergenic regions
Euchromtain
Coding sequences
Heterochromatin
Early Rings
Late Rings
Trophozoites
Schizonts
log2 (H3.3/H3core)
Sub. rep.
CS
IG
r = 0.4
r = 0.071
r = 0.5
r= 0.15
r = -0.066
r = 0.39
r = 0.014
r = -0.08
r = 0.3
r = 0.37
r = 0.094
r = 0.47
GC [%]
(B)
Euchromatin
Euchromatin
Intergenic regions
Euchromtain
Coding sequences
Heterochromatin
Schizonts
log2 (H3.3/H3core)
Sub. rep.
CS
IG
r = 0.21
r = 0.075
r = 0.4
GC [%]

Fig. S6

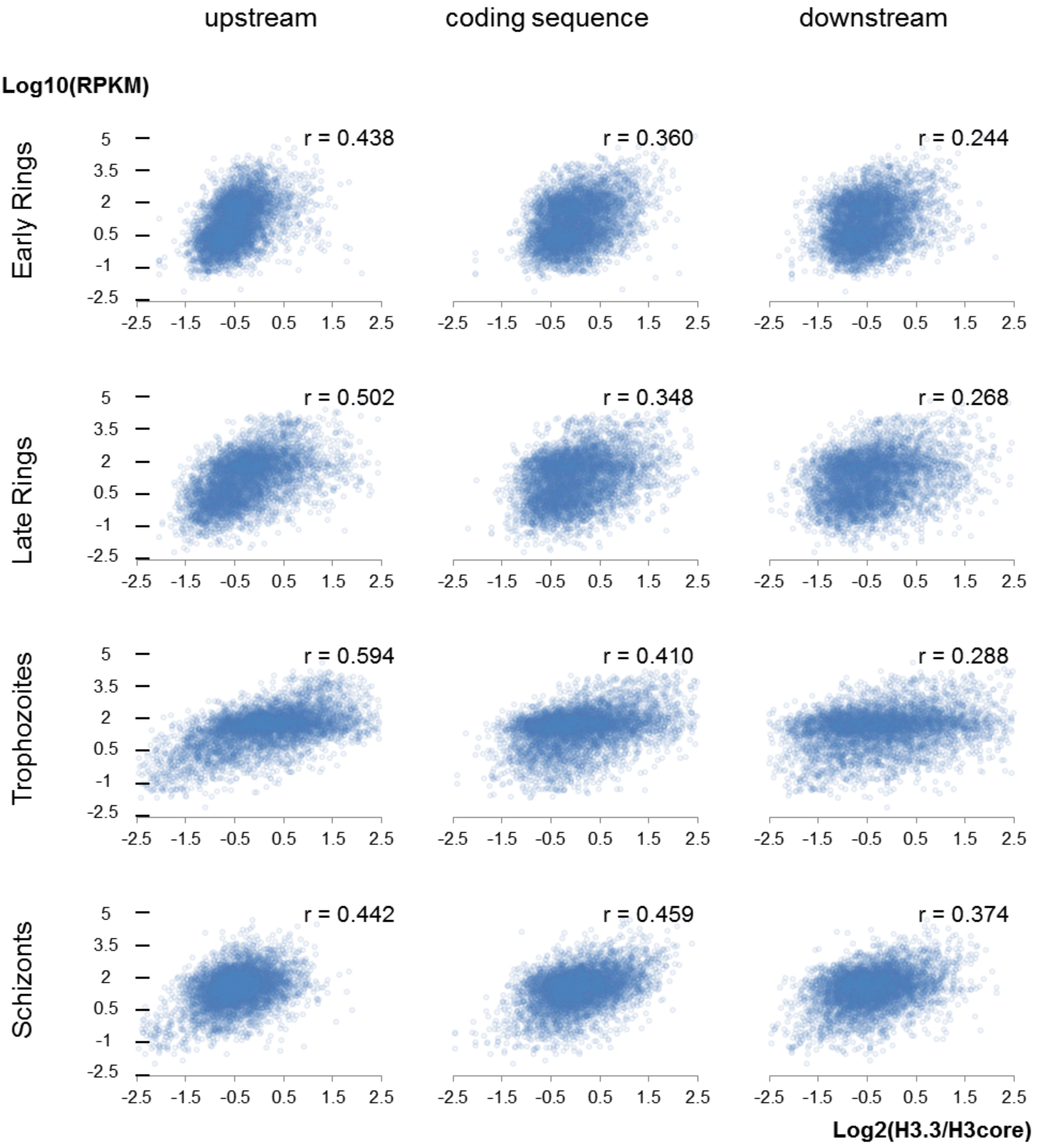

upstream
coding sequence
downstream
Log10(RPKM)
Early Rings
Late Rings
Trophozoites
Schizonts
r = 0.438
r = 0.360
r = 0.244
r = 0.502
r = 0.348
r = 0.268
r = 0.594
r = 0.410
r = 0.288
r = 0.442
r = 0.459
r = 0.374
5
3.5
2
0.5
-1
-2.5
-2.5
-1.5
-0.5
0.5
1.5
2.5
Log2(H3.3/H3core)

Fig. S7

(A)

var2CA (Pf3D7_1200600) selection for two clones

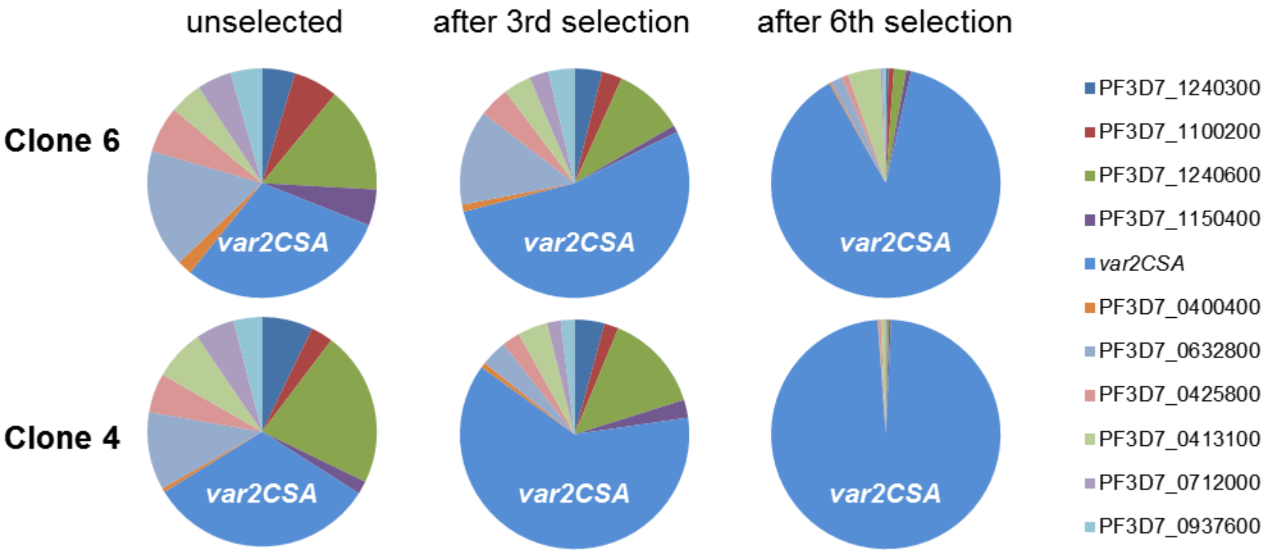

(B)

PF3D7_0223500 expression after Blasticidin treatment

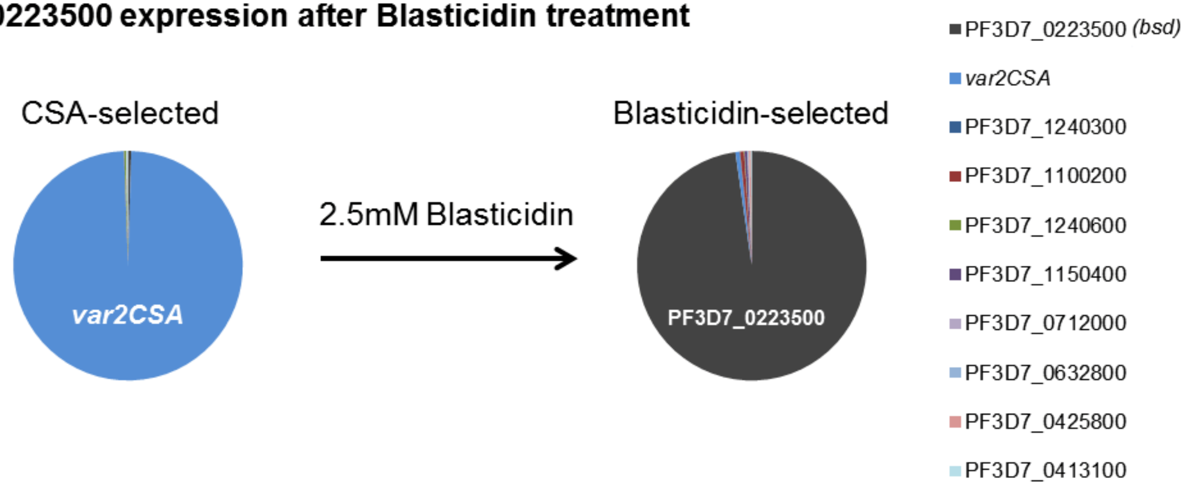

Supplement: Supplementary Information [file srep31965-s1.pdf]
